# Macrophages in rosacea: pathogenesis and therapeutic potential

**DOI:** 10.3389/fimmu.2025.1595493

**Published:** 2025-07-31

**Authors:** Xiaolin Wang, Huanyu Shi, Xueli Li, Yanyan Feng

**Affiliations:** Department of Dermatology, Chengdu Second People’s Hospital, Chengdu, Sichuan, China

**Keywords:** macrophages, rosacea, inflammatory skin disease, pathogenesis, treatment

## Abstract

**Objective:**

Rosacea is a chronic inflammatory dermatosis predominantly affecting the central face, with its pathogenesis not yet fully elucidated. Macrophages, as innate immune cells in the human body, play a crucial role in inflammatory responses. However, the specific mechanistic role of macrophages in rosacea remains incompletely understood. This review aims to comprehensively analyze and discuss the functions of macrophages, their involvement in the pathogenesis of rosacea, and their potential as therapeutic targets.

**Method:**

A systematic literature search was conducted using keywords such as “rosacea” and “macrophage” in databases including PubMed and Web of Science, without restrictions on article type or publication date, to ensure a comprehensive retrieval of relevant studies. Additionally, the references cited in the retrieved articles were manually searched to gather further pertinent knowledge.

**Results:**

For the articles obtained from the database searches, we focused solely on those that mentioned the role of macrophages in rosacea and related therapeutic approaches to ensure the accuracy of the content. Ultimately, 121 articles were selected for inclusion in this review, encompassing review articles, original research studies, meta-analyses, and other types of publications.

**Conclusion:**

This review summarizes the latest research progress on the role of macrophages in the pathogenesis of rosacea, emphasizing their significant involvement through the regulation of immune responses, angiogenesis, oxidative stress, fibrosis, and other processes. Furthermore, the potential of macrophages as therapeutic targets for rosacea is explored, which warrants further investigation in the future. Despite the advancements made, numerous unresolved questions remain regarding the mechanistic role of macrophages in rosacea. Future research is imperative to delve deeper into the underlying mechanisms, thereby providing novel insights into the pathogenesis and treatment of rosacea.Please confirm that the below Frontiers AI generated Alt-Text is an accurate visual description of your Figure(s). These Figure Alt-text proposals won't replace your figure captions and will not be visible on your article. If you wish to make any changes, kindly provide the exact revised Alt-Text you would like to use, ensuring that the word-count remains at approximately 100 words for best accessibility results. Further information on Alt-Text can be found here.

## Graphical Abstract

Macrophages participate in the pathogenesis and progression of rosacea through multiple mechanisms, including immune responses, angiogenesis, oxidative stress, and fibrosis, thereby playing a pivotal role in the disease’s development. Future research could explore novel therapeutic strategies targeting macrophages for the management of rosacea. The figure was created with Figdraw.

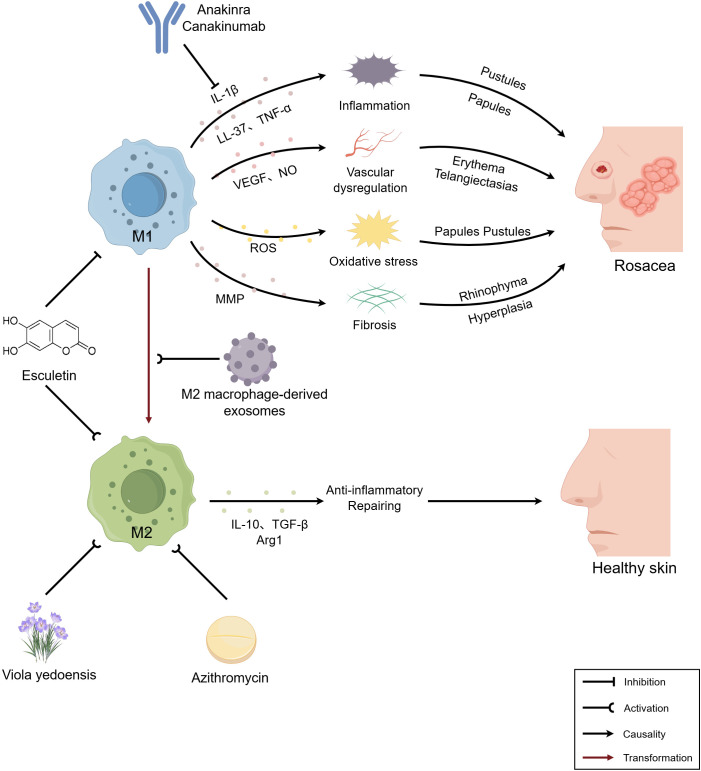

## 1 Introduction

Rosacea is a prevalent chronic inflammatory skin disease predominantly affecting the midface, clinically manifested by episodic flushing, persistent erythema, telangiectasia, papules, and pustules, with or without hypertrophy and hyperplasia (1). Additionally, patients often experience sensations of stinging and burning, significantly impacting their social interactions and psychological well-being (2). Based on the differences in clinical features, it can be classified into four subtypes: erythematotelangiectatic rosacea (ETR), papulopustular rosacea (PPR), phymatous rosacea (PHR), and ocular rosacea (OR) (3). The etiology and pathogenesis of rosacea have not been fully elucidated; It is currently believed to be associated with genetic susceptibility, immune dysregulation, and neurovascular dysfunction, among others (4, 5). Exogenous stimuli such as ultraviolet (UV) radiation, alcohol, microbial flora, and psychological stress can also induce or exacerbate the symptoms of the disease (6, 7). Macrophages, as crucial effector cells of the innate immune system, have been demonstrated to play a significant role in rosacea. This article reviews the recent research progress of macrophages in rosacea.

## 2 Classification and functions of macrophages

Macrophages are innate immune cells differentiated and developed from circulating monocytes, widely distributed throughout various tissues of the body, and constitute the first line of defense against pathogens in humans (8). They can be classified into tissue-resident and migratory subsets. The traditional view holds that tissue-resident macrophages originate from circulating monocytes. However, recent studies have indicated that the majority of tissue-resident macrophages actually derive from the yolk sac and fetal liver during embryonic development (9), primarily maintaining local tissue homeostasis, whereas the migratory macrophages primarily assist in host defense and pathological signal transduction (10).

When circulating monocytes migrate to tissues, they can differentiate and develop into two macrophage subpopulations with different functions under the stimulation of various signaling factors in the microenvironment: classically activated macrophages (M1) and alternatively activated macrophages (M2) (11). M1 macrophages differentiate through the binding of Toll-like receptors (TLRs) on the monocyte surface to microbes and their products or via induction by Th1-type cytokines such as interferon (IFN)-γ and tumor necrosis factor (TNF)-α. They specifically express markers such as CD40, CD80, and CD86 on their surface and exert pro-inflammatory effects by secreting pro-inflammatory cytokines such as interleukin (IL)-1β, IL-6, and TNF-α, as well as chemokines such as C-C motif chemokine ligand (CCL) 2, CCL3, and IL-8, thereby eliminating invading pathogens and initiating and maintaining inflammatory responses (11–13). In contrast, M2 macrophages differentiate from monocytes under the induction of Th2-type cytokines such as IL-4 and IL-13. They specifically express markers such as CD163, CD204, and CD206 on their surface and inhibit inflammatory responses by secreting anti-inflammatory cytokines such as IL-10, transforming growth factor (TGF)-β, vascular endothelial growth factor (VEGF), and arginase 1 (Arg1), participating in tissue repair and wound healing during the later stages of inflammation (11–14). In fact, M1 and M2 are considered to be the two extremes of a continuous spectrum of macrophage functional states and do not represent all states of macrophages. Individual macrophages simultaneously expressing both M1-type and M2-type markers can be observed *in vivo* (15). Therefore, macrophages exhibit a high degree of plasticity, with M1 and M2 being able to interconvert in response to changes in the local microenvironment. During the wound healing process, as M1 macrophages phagocytose necrotic cellular debris, local pro-inflammatory signals diminish. IL-4 and IL-13 promote the conversion of M1 to M2 through the signal transducer and activator of transcription (STAT) 6 and peroxisome proliferator-activated receptor (PPAR) γ signaling pathways, thereby limiting inflammation and facilitating healing (16, 17). Correspondingly, activation of the TLR3/IFN-αβ signaling pathway can induce the conversion of M2 to M1, successfully reversing and controlling tumor growth (18). Luo et al. (19) demonstrated that folate-targeted Toll-like receptor 7 agonist (FA-TLR7-1A) can reprogram immunosuppressive M2 macrophages into pro-inflammatory M1 macrophages by targeted activation of the TLR7 signaling pathway, thereby breaking the immunosuppressive state of the tumor microenvironment and enhancing the efficacy of CAR-T cells. Therefore, maintaining the dynamic balance of M1/M2 polarization is crucial for correcting immune imbalance and promoting disease resolution.

Macrophages in the skin are primarily distributed within the dermis, where they collaborate with endothelial cells, neutrophils, mast cells, and other cellular components to regulate skin homeostasis and inflammatory responses (20). These macrophages are implicated in the pathogenesis and progression of various inflammatory skin diseases, including rosacea, psoriasis, and atopic dermatitis, among others (21–23).

## 3 The role of macrophages in rosacea

Buhl et al. (24) found that in patients with ETR, PPR, and PHR, the number of macrophages in skin lesions was significantly higher than in healthy controls, suggesting the involvement of macrophages in the pathogenesis of rosacea. Subsequent studies further confirmed that M1 macrophages, rather than M2 macrophages, are highly infiltrated in the lesion areas of rosacea (25–27), and their infiltration level is positively correlated with Clinical Erythema Assessment (CEA) and Investigator’s Global Assessment (IGA) scores, and depletion of M1 macrophages can significantly reduce skin inflammation (26). Therefore, macrophages play a key role in the pathogenesis of rosacea. This article elaborates on their functions and mechanisms of action in rosacea.

### 3.1 Macrophages participate in the immuno-inflammatory response of rosacea

**Figure 1:**
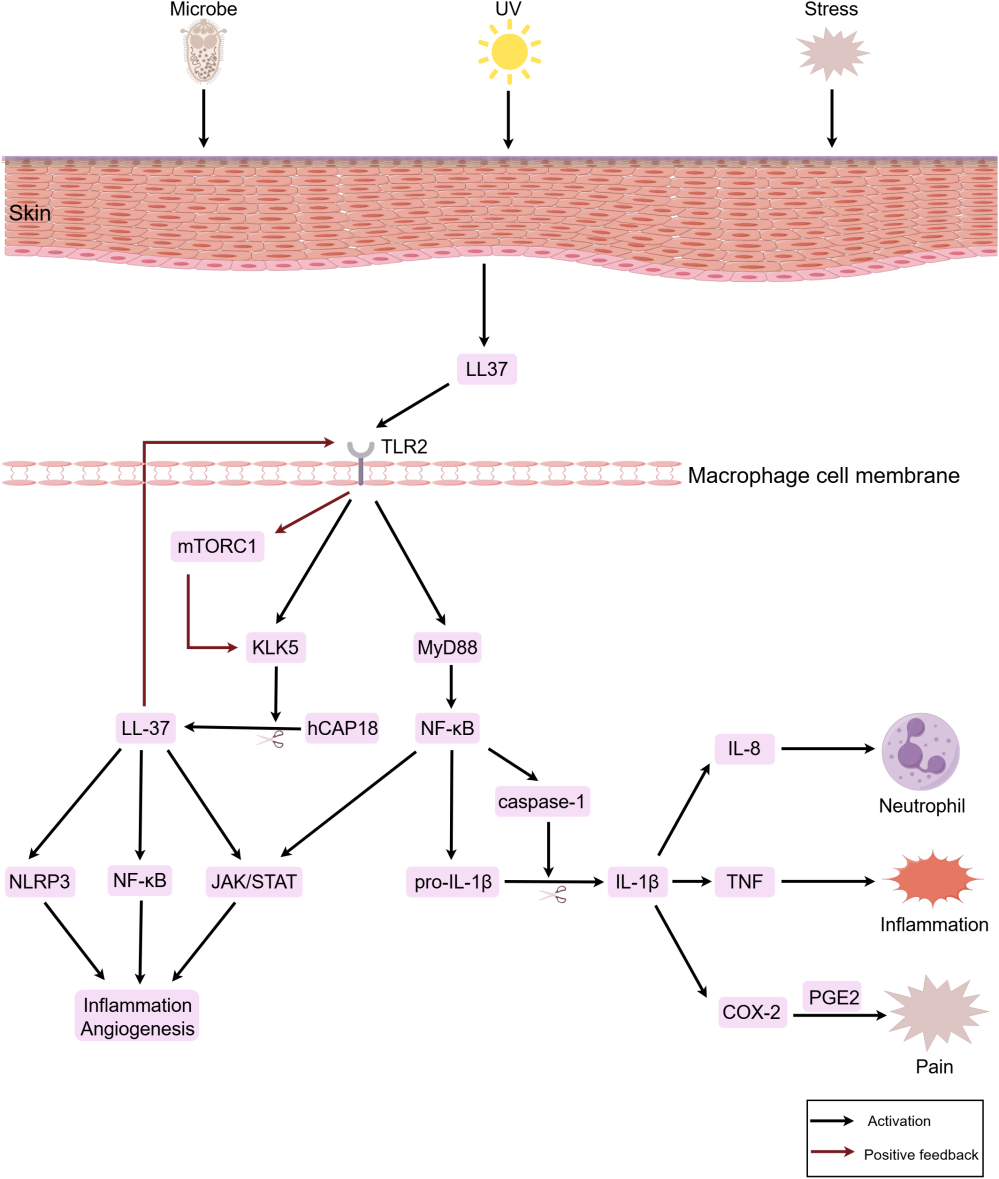
Macrophages participate in the immuno-inflammatory response of rosacea. When the skin is stimulated by factors such as microorganisms, ultraviolet radiation, and psychological stress, keratinocytes are activated and release LL-37. This subsequently activates TLR2 on macrophages. Through signal transduction, downstream signaling pathways including JAK/STAT, NF-κB, and NLRP3 are activated, thereby inducing inflammatory responses and vasodilation. In the figure, LL-37, IL-1β, IL-8, TNF, and PGE2 are downstream factors secreted by macrophages. In the figure, the red arrows denote positive feedback loops, which continuously amplify the inflammatory response. The figure was created with Figdraw.

Studies indicate that macrophages participate in the pathogenesis of rosacea by eliciting inflammation associated with innate immune responses. When the skin is stimulated by factors such as microorganisms and their products, ultraviolet radiation, and psychological stress, keratinocytes become activated and release LL-37, which in turn activates TLR2 on macrophages, upregulating the expression of kallikrein-related peptidase 5 (KLK5). Subsequently, KLK5 cleaves hCAP18 into its active form, LL-37 (28, 29). LL-37 not only directly opposes invading pathogens (30), but also activates signaling pathways such as janus kinase (JAK)/STAT, nuclear factor-kappa B (NF-κB), and NOD-like receptor family pyrin domain containing 3 (NLRP3), thereby promoting the production of pro-inflammatory cytokines like IL-1β, IL-6, and TNF-α, inducing and maintaining inflammatory responses and angiogenesis (31–33). Concurrently, LL-37 produced by macrophages binds to TLR2 through an autocrine mechanism, activating the mammalian target of rapamycin complex 1 (mTORC1) pathway. This leads to an increased production of LL-37 and amplification of the inflammatory response, forming a positive feedback loop (34). Additionally, macrophages upregulate the gene expression of NLRP3 and pro-IL-1β via the TLR2/myeloid differentiation primary response gene 88 (MyD88)/NF-κB pathway, facilitating the recruitment and activation of cysteine-aspartic acid protease-1 (caspase-1), which induces pyroptosis and the release of IL-1β and IL-18 (35, 36). As a pivotal inflammatory cytokine, IL-1β upregulates the expression of cytokines such as IL-8, TNF, and cyclooxygenase (COX)-2 (37). Among these, IL-8 promotes pustule formation in PPR skin lesions by mediating neutrophil chemotaxis, TNF mediates inflammatory cascades, promoting papule formation and the sensation of burning, and COX-2 catalyzes the production of prostaglandin (PG)E2, inducing pain (38–40) (**Figure 1**).

Studies have found that there is a significant infiltration of CD4+ T cells in the skin lesions of patients with rosacea, predominantly comprising helper T cells (Th) 1 and Th17 cells, suggesting that adaptive immune responses also play a role in the pathogenesis of rosacea (24, 41). Macrophages, as professional antigen-presenting cells, process and present antigens to T cells by phagocytosis or recognition of microbes and their products through TLRs, inducing the occurrence of adaptive immune responses (42). Concurrently, M1 macrophages facilitate the activation and proliferation of T cells through the interaction of their surface markers CD40, CD80, and CD86 with co-stimulatory molecules on the surface of T cells (43). Furthermore, IL-12 secreted by macrophages induces the differentiation of Th0 cells into Th1 cells (44, 45), while IL-1β and IL-6 induce the differentiation of Th0 cells into Th17 cells (46, 47). Correspondingly, Th1 cells stimulate the polarization of macrophages towards the M1 phenotype by secreting type 1 inflammatory cytokines such as IFN-γ and TNF-α (48). Th17 cells, on the other hand, secrete IL-17 to stimulate local tissue cells to produce chemokines, recruiting monocytes and neutrophils to the lesion site, thereby exacerbating local inflammation (49, 50). Therefore, in the pathogenesis of rosacea, a positive feedback loop forms between the innate immune system represented by macrophages and the adaptive immune system mediated by CD4+ T cells, continuously amplifying the immune inflammatory response and promoting disease progression.

### 3.2 Macrophages participate in vascular dysregulation in rosacea

Studies found that under inflammatory conditions, M1 macrophages activate STAT3 and NF-κB signaling pathways through an IL-1β autocrine loop. In an IL-1β-dependent manner, they bind to the nuclear VEGFA promoter, thereby promoting the transcription and expression of VEGFA and participating in angiogenesis (51, 52). Additionally, pro-inflammatory cytokines such as IL-1, IL-6, and TNF-α released by macrophages can also upregulate the expression of VEGF (53). Among them, IL-6 induces corneal fibroblasts to produce VEGF by activating the STAT3 signaling pathway, stimulating corneal neovascularization, and participating in the occurrence and development of OR (31, 54). Concurrently, VEGF induces endothelial cells to produce chemokines such as monocyte chemotactic protein (MCP)-1 and IL-8, as well as adhesion molecules such as intercellular adhesion molecule (ICAM) and vascular cell adhesion molecule (VCAM), recruiting and activating inflammatory cells such as the monocytic macrophage lineage and neutrophils, amplifying the inflammatory response (55–57). Therefore, VEGF is also considered a key molecule linking immune inflammation and angiogenesis.

Furthermore, M1 macrophages specifically express inducible nitric oxide synthase (iNOS), which catalyzes the conversion of arginine to nitric oxide (NO) to defend against invading pathogens (58, 59). Concurrently, NO enters vascular smooth muscle cells and binds to soluble guanylyl cyclase (sGC), activating it and promoting the production of cyclic guanosine monophosphate (cGMP) (60). As a second messenger, cGMP activates cGMP-dependent protein kinase G (PKG). PKG then phosphorylates downstream effector proteins, reducing the intracellular concentration of free Ca^2+^. This, in turn, inhibits vascular smooth muscle cell proliferation and induces their relaxation, leading to local vasodilation and an increased blood flow (61, 62). This may be associated with the clinical manifestation of facial erythema in patients with rosacea. Additionally, vasodilatation and increased permeability of newly formed capillaries lead to plasma extravasation, forming local redness and swelling, while facilitating the infiltration of more inflammatory cells and mediators into the lesion site, exacerbating local inflammation. In summary, in rosacea, angiogenesis and inflammatory responses regulate and promote each other, jointly driving disease progression.

### 3.3 Macrophages participate in oxidative stress in rosacea

Oxidative stress represents a pathological state of imbalance between oxidation and antioxidant defenses within the organism, leading to the excessive production of reactive oxygen species (ROS) (63). ROS are oxygen-derived reactive molecules with unpaired electrons that can induce cellular stress and damage (64, 65). Demir et al. (66) found that the thiol-disulfide homeostasis (TDH) in the serum of patients with rosacea shifts towards disulfides, indicating the presence of oxidative stress. Traditionally, neutrophils have been considered the primary source of ROS in rosacea (67–69), but recent studies suggest that macrophages may also be involved, jointly mediating oxidative stress in this disorder. Macrophages bind to bacterial lipopolysaccharide (LPS) through TLR4, prompting the Toll-IL-1R (TIR) domain of TLR4 to interact with the carboxyl terminus of the intracellular nicotinamide adenine dinucleotide phosphate (NADPH) oxidase. This interaction catalyzes the transfer of electrons from NADPH to O_2_, leading to the generation of O_2_^-^. O_2_^-^ serves as a common precursor for all ROS subspecies generated within cells. It rapidly undergoes dismutation to form hydrogen peroxide (H_2_O_2_), which subsequently reacts to generate additional ROS, contributing to the elimination of pathogens (70, 71). Simultaneously, ROS acts as a second messenger to activate downstream mitogen-activated protein kinase (MAPK) and NF-κB signaling pathways, further regulating cytokine expression and amplifying the immune response (72, 73). Additionally, macrophage-derived TNF-α induces the production of mitochondrial ROS through TNF receptor 1 (TNFR1), which in turn inhibits the phosphatase activity of c-Jun N-terminal kinase (JNK), leading to sustained activation of the JNK pathway and promoting cell apoptosis and necrosis (74, 75).

However, excessive ROS can damage various skin cells, including keratinocytes, fibroblasts, and endothelial cells, prompting the release of pro-inflammatory cytokines such as IL-1 and TNF-α, and causing oxidative damage to the extracellular matrix (31, 76, 77), thereby disrupting skin barrier function. Additionally, ROS accumulate within macrophages, forming advanced oxidation protein products (AOPP), which leads to protein peroxidation and subsequently affects the normal structure and function of cells (78, 79). Furthermore, ROS can combine with NO catalyzed by iNOS to form peroxynitrite (ONOO^-^), which damages DNA through oxidative deamination, resulting in impaired macrophage function (58, 80). Based on this, we speculate that when macrophages are damaged or die due to oxidative stress, they may release more inflammatory cytokines, further exacerbating the inflammatory response. This hypothesis needs to be validated through experimental research (**Table 1**).

**Table 1:** Mechanisms of macrophage involvement in oxidative stress in rosacea.

Substance	Mechanism of action	Outcome
TLR4 (70–73)	Its intracellular TIR domain binds to NADPH oxidase, catalyzing the production of a substantial amount of ROS.	Intracellularly: activation of downstream MAPK and NF-κB pathways. Extracellularly: disruption of various skin cells and the extracellular matrix.
TNF-α (74, 75)	Binds to TNFR1 to induce the generation of mitochondrial ROS.	Sustained activation of the JNK pathway promoting apoptosis and necrosis.
AOPP (78, 79)	Leads to protein peroxidation.	Disruption of normal cellular structure and function.
ONOO- (58, 80)	Damages DNA through oxidative deamination.	Disruption of cellular function.

### 3.4 Macrophages participate in metabolic dysregulation in rosacea

Recent studies have indicated that patients with rosacea often exhibit metabolic dysfunction and may even coexist with metabolic diseases such as obesity, hypertension, and hypercholesterolemia (81–83). Metabolic abnormalities in rosacea have gradually become a research focus, and macrophages potentially play a role in this process. M1 macrophages dominate the inflammatory response and undergo metabolic reprogramming, shifting their glucose metabolism from oxidative phosphorylation to glycolysis, which facilitates the rapid cellular response to local infection or inflammation (84, 85). However, the blockade of the tricarboxylic acid cycle leads to the accumulation of intermediate metabolites such as citrate and succinate. Citrate can induce macrophages to produce inflammatory mediators such as NO, ROS, and PG, whereas succinate stabilizes hypoxia-inducible factor-1α(HIF-1α) by inhibiting prolyl hydroxylase domain (PHD) enzymes, thereby promoting the transcription of IL-1β (86, 87). Collectively, these effects synergistically enhance the inflammatory response. As mentioned earlier, arginine in M1 macrophages is catalyzed by iNOS to produce NO and citrulline. NO not only dilates blood vessels but also, when combined with ROS to form reactive nitrogen species, can inactivate the mitochondrial electron transport chain, thereby preventing the repolarization of M1 to M2 (88, 89). This mechanism may explain the chronic and relapsing nature of rosacea, which is often difficult to cure.

However, the specific mechanisms underlying the role of macrophages in metabolic abnormalities associated with rosacea remain unclear. Tang et al. (90) found in their study using an *in vitro* acne disease model that M1 macrophages can promote lipid synthesis in sebocytes, significantly increasing sebum accumulation. Current research on sebaceous gland metabolism in rosacea has shown a decrease in the levels of long-chain saturated fatty acids in sebum, with no change in the total amount of sebum secreted (91). This suggests that the regulatory mechanisms of macrophages in sebaceous gland metabolism in rosacea differ from those in acne. Further research is needed to uncover the underlying mechanisms of their potential roles.

### 3.5 Macrophages participate in fibrosis in rosacea

In the advanced stages of PHR, skin fibrosis occurs, during which M1 macrophages secrete matrix metalloproteinases (MMP) to degrade the extracellular matrix (ECM) (92). This process aids in the removal of damaged or necrotic tissues and creates space for the influx of new cells and the deposition of provisional ECM, thereby initiating ECM remodeling (93, 94). Additionally, M1 macrophages promote the proliferation and activation of fibroblasts through IL-1β, upregulating the expression of type I and III collagens, as well as fibronectin, leading to excessive ECM deposition and fibrosis (95). Correspondingly, activated fibroblasts release macrophage colony-stimulating factor 1 (CSF1), CCL2, and IL-6, which recruit and activate monocytes and macrophages (96, 97). Furthermore, IL-6, secreted by M1 macrophages, acts on fibroblasts to further promote fibrosis (98). However, previous studies have demonstrated that M2 macrophages play a pivotal role in fibrosis. M2 macrophages facilitate ECM deposition and remodeling by producing ECM components such as fibronectin and collagen (99). They also stimulate fibroblasts to generate a series of ECM proteins through the release of cytokines, including platelet-derived growth factor (PDGF) and TGF-β (100, 101). Additionally, M2 macrophages contribute to tissue fibrosis by modulating the activity of ECM-remodeling enzymes (102, 103). However, the mechanism of action of M2 macrophages in fibrotic lesions is more complex. In the fibrosis of organs such as the liver, kidneys, and lungs, an increased proportion of M2 macrophages, which is positively correlated with the degree of fibrosis, has been observed, along with functional dysregulation (104–106). For instance, in renal fibrosis, M2 macrophages undergo proliferation-dependent phenotypic switching induced by the overexpression of CSF1 in renal tubular epithelial cells, resulting in a functional transition from anti-inflammatory repair to profibrotic activity (107). Shen et al. (108) demonstrated that M2 macrophages secrete excessive TGF-β1, serving as the primary source of TGF-β1 in renal fibrosis, directly inducing epithelial-mesenchymal transition (EMT) in renal tubular epithelial cells. Additionally, under continuous stimulation by excessive TGF-β1, M2 macrophages undergo macrophage-to-myofibroblast transition (MMT) via the TGF-β1/Smad3 signaling pathway, secreting large amounts of collagen and thereby leading to ECM accumulation and exacerbating fibrosis progression (109, 110). Lv et al. (111) revealed that the number of M2 macrophages in the skin lesions of patients with keloids is higher than that in normal skin, with an elevated M2/M1 ratio. Moreover, during keloid formation, M2 macrophages exhibit profibrotic functions, continuously secreting TGF-β1, which activates the Smad2/3 signaling pathway in fibroblasts, induces collagen synthesis and the expression of MMP inhibitors, and creates an irreversible fibrotic microenvironment (112, 113). We hypothesize that M2 macrophages may be involved in the fibrosis observed in the late stages of PHR. However, no studies have yet demonstrated whether there are differences in the expression of M2 macrophages between rosacea and normal skin, or whether there is functional dysregulation of M2 macrophages. Further research is needed to verify these aspects in the future.

## 4 Targeting macrophages for the treatment of rosacea

Based on the pivotal role of M1 macrophages in rosacea, inhibitors targeting their pro-inflammatory cytokines exhibit promising therapeutic potential. For example, IL-1β pathway inhibitors (e.g. anakinra and canakinumab) alleviate skin inflammation by blocking the binding of IL-1β to its receptor, thereby inhibiting downstream signaling (114, 115). In recent years, the anti-inflammatory properties of M2 macrophages have offered a new direction for the treatment of inflammatory skin diseases. Studies have shown that azithromycin activates M2 macrophages via the phosphatidylinositol 3-kinase (PI3K)/protein kinase B (AKT) signaling pathway, alleviating symptoms of systemic lupus erythematosus (116); additionally, the traditional Chinese medicine Viola yedoensis promotes M2 macrophage polarization by activating the JAK2/STAT3 signaling pathway, improving symptoms of atopic dermatitis (117). However, persistent M1 macrophages without conversion to M2 macrophages can lead to prolonged disease progression. Research by Wang et al. (118) has demonstrated that M2 macrophage-derived exosomes induce reprogramming of M1 macrophages into M2 macrophages through the PI3K/AKT signaling pathway, improving the immune microenvironment and accelerating diabetic fracture healing. Furthermore, Esculetin modulates metabolic reprogramming by inhibiting glycolysis in M1 macrophages and promoting fatty acid β-oxidation in M2 macrophages, thereby balancing M1/M2 macrophage polarization and alleviating sepsis-induced lung injury (119). Therefore, regulating macrophage phenotypic conversion, maintaining the M1/M2 macrophage balance, and promoting inflammation resolution and tissue repair represent potential new strategies for the treatment of rosacea (**Table 2**).

**Table 2:** Potential therapeutic interventions targeting macrophages for the management of rosacea.

Treatment	Mechanism of action	Treatment efficacy	Application status
IL-1β pathway inhibitors (e.g., anakinra and canakinumab) (104, 115)	Blocks the binding of IL-1β to its receptor.	Significant reduction in inflammatory lesion count and erythema.	Hidradenitis Suppurativa, Psoriasis Arthritis, Pyoderma Gangrenosum et al.
Azithromycin (116)	Activates the PI3K/AKT signaling pathway to thereby activate M2 macrophage.	Reduced pro-inflammatory cytokines, elevated anti-inflammatory cytokines, and alleviated SLE symptoms.	Cystic fibrosis, Chronic Obstructive Pulmonary Disease, Spinal cord injury et al.
Viola yedoensis (117)	Activates the JAK2/STAT3 signaling pathway to promote M2 macrophage polarization.	Reduction in clinical scoring and epidermal thickness.	Atopic dermatitis.
M2 macrophage-derived exosomes (118)	Activates the PI3K/AKT signaling pathway to facilitate M1 reprogramming into M2 macrophage.	Improved immune microenvironment, accelerated healing of damaged tissues.	Diabetic fracture.
Esculetin (119)	Regulates metabolic reprogramming to balance M1/M2 macrophage polarization.	M1/M2 macrophage polarization balance restored, inflammatory response attenuated.	Sepsis‐induced acute lung injury.

## 5 Conclusion

**Figure 2:**
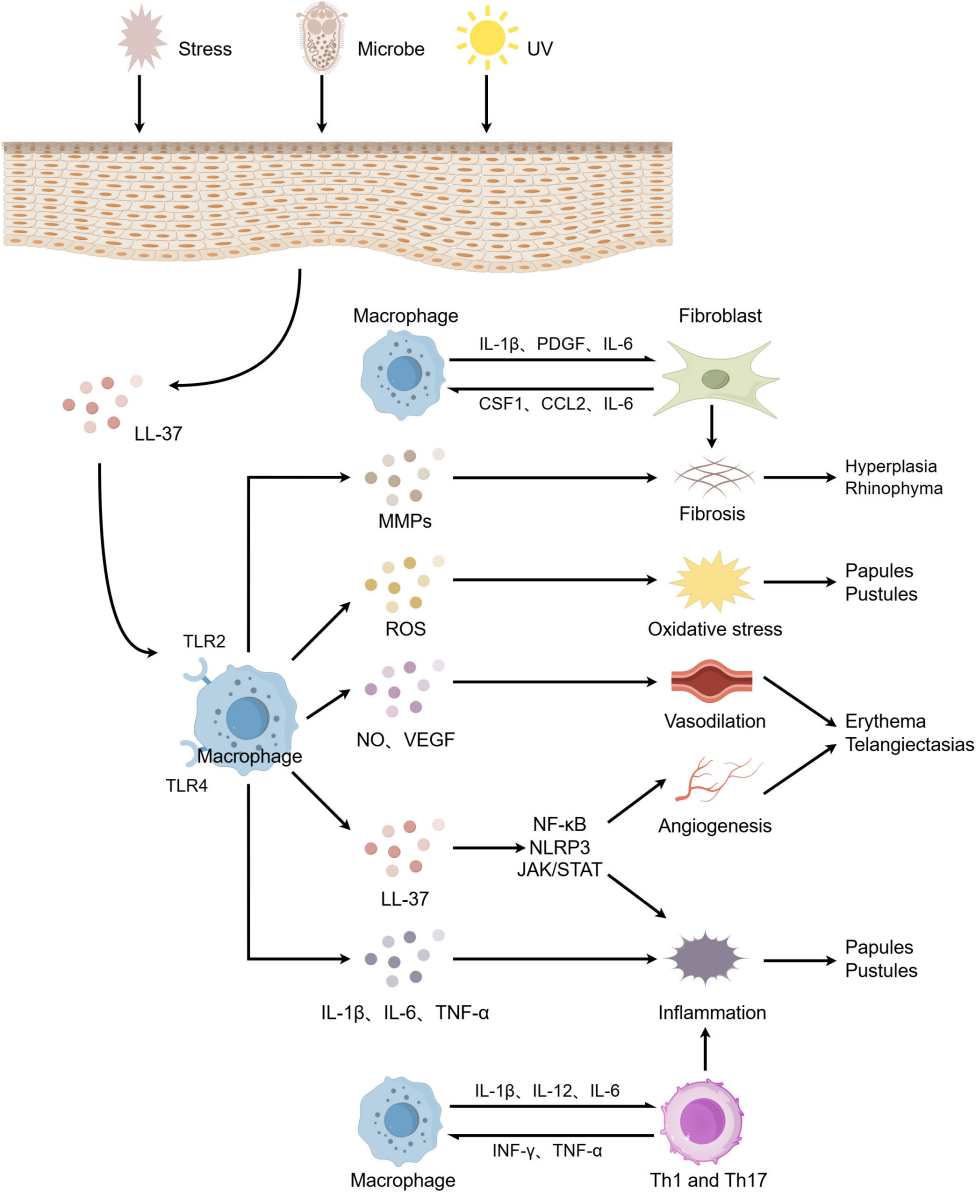
The role of macrophages in the pathogenesis of rosacea. When the skin is stimulated by factors such as microorganisms, ultraviolet radiation, and psychological stress, keratinocytes release LL-37, which activates macrophages. These activated macrophages subsequently release a cascade of pro-inflammatory cytokines and inflammatory mediators, promoting inflammatory responses, vasodilation and angiogenesis, oxidative stress, and fibrosis. These processes collectively lead to clinical manifestations including papules and pustules, erythematosus plaques, telangiectasia, and rhinophyma. Additionally, macrophages interconnect innate and adaptive immunity through bidirectional induction with Th1/Th17 cells, exacerbating the inflammatory process. Furthermore, the interplay between macrophages and fibroblasts further promotes fibrosis. The figure was created with Figdraw.

In this review, we comprehensively elucidate the pivotal role of macrophages in the pathogenesis of rosacea (**Figure 2**). Macrophages participate in and drive the onset and progression of rosacea through various mechanisms, including the modulation of immune responses, angiogenesis, oxidative stress, and metabolic disturbances. However, numerous unresolved questions persist in current research. For instance, the interplay between macrophages and other immune cells, such as neutrophils and mast cells, has not been fully elucidated, and the specific regulatory mechanisms of macrophages in metabolic abnormalities require further exploration.

Furthermore, we also discuss the therapeutic prospects of targeting macrophages in the treatment of rosacea. M1 macrophages are implicated in multiple aspects of rosacea pathogenesis, whereas M2 macrophages exhibit anti-inflammatory and tissue repair functions. Therefore, modulating the M1/M2 macrophage balance may represent a novel therapeutic direction for rosacea. However, this balance is not a simple phenotypic conversion but rather maintained at a dynamic level to prevent excessive activation of M2 macrophages, which could lead to tissue fibrosis or even scar formation.

In summary, future research needs to continually delve into the mechanisms underlying the role of macrophages in rosacea, with the aim of unveiling the molecular networks involved in the onset and progression of the disease. This endeavor will provide theoretical foundations and novel strategies for the treatment of rosacea. Finally, we summarized the most recent research advancements on macrophages in rosacea over the past five years (**Table 3**).

**Table 3:** Summary of the latest research on macrophages in rosacea over the past five years.

Latest research	Conclusion	Year
Vascular dysregulation (51, 52)	M1 macrophages activate the STAT3 and NF-κB signaling pathways through an IL-1β autocrine loop, leading to the release of VEGF and subsequent promotion of angiogenesis.	2022, 2023
Oxidative stress (71)	Upon binding of TLR4 on macrophages to LPS, its intracellular TIR domain interacts with NADPH oxidase to catalyze the generation of a large amount of ROS.	2021
Oxidative stress (58, 80)	ROS and NO generated by macrophages combine to form ONOO−, inducing DNA damage via oxidative deamination and impairing macrophage function.	2020
Metabolic dysregulation (88, 89)	NO and reactive nitrogen species from M1 macrophages inactivate mitochondrial electron transport, blocking M1-to-M2 repolarization and potentially prolonging disease.	2023, 2025
Case report (120)	The JAK1 inhibitors upadacitinib and abrocitinib may be promising medical options for patients with refractory rosacea.	2024
Case report (121)	Chronic and persistent inflammation around the isthmus produced in scalp rosacea may form peripilar scaling resembling that found in lichen planopilaris.	2021

## Glossary

ETR: erythematotelangiectatic rosacea
PPR: papulopustular rosacea
PHR: phymatous rosacea
OR: ocular rosacea
UV: ultraviolet
M1: classically activated macrophages
M2: alternatively activated macrophages
TLR: Toll-like receptor
IFN-γ: interferon-γ
TNF-α: tumor necrosis factor-α
IL: interleukin
CCL: C-C motif chemokine ligand
TGF-β: transforming growth factor-β
VEGF: vascular endothelial growth factor
Arg1: arginase 1
STAT: signal transducer and activator of transcription
PPARγ: peroxisome proliferator-activated receptor γ
FA-TLR7-1A: folate-targeted Toll-like receptor 7 agonist
CEA: Clinical Erythema Assessment
IGA: Investigator's Global Assessment
KLK5: kallikrein-related peptidase 5
JAK: Janus Kinase
NF-κB: nuclear factor-kappa B
NLRP3: NOD-like receptor family pyrin domain containing 3
mTORC1: mammalian target of rapamycin complex 1
MyD88: myeloid differentiation primary response gene 88
caspase-1: cysteine-aspartic acid protease-1
COX-2: cyclooxygenase-2
PGE2: prostaglandin E2
Th: helper T cells
MCP-1: monocyte chemotactic protein-1
ICAM: intercellular adhesion molecule
VCAM: vascular cell adhesion molecule
iNOS: inducible nitric oxide synthase
NO: nitric oxide
sGC: soluble guanylyl cyclase
cGMP: cyclic guanosine monophosphate
PKG: protein kinase G
ROS: reactive oxygen species
TDH: thiol-disulfide homeostasis
LPS: bacterial lipopolysaccharide
TIR: Toll-IL-1R
NADPH: nicotinamide adenine dinucleotide phosphate
H2O2: hydrogen peroxide
MAPK: mitogen-activated protein kinase
TNFR1: tumor necrosis factor receptor 1
JNK: c-Jun N-terminal kinase
AOPP: advanced oxidation protein products
ONOO-: peroxynitrite
HIF-1α: hypoxia-inducible factor-1α
PHD: prolyl hydroxylase domain
MMP: matrix metalloproteinases
ECM: extracellular matrix
PDGF: platelet-derived growth factor
CSF1: macrophage colony-stimulating factor 1
EMT: epithelial-to-mesenchymal transition
MMT: macrophage-to-myofibroblast transition
PI3K/AKT: phosphatidylinositol 3-kinase/protein kinase B
